# A Temperature-Controlled Patch Clamp Platform Demonstrated on Jurkat T Lymphocytes and Human Induced Pluripotent Stem Cell-Derived Neurons

**DOI:** 10.3390/bioengineering7020046

**Published:** 2020-05-22

**Authors:** Jann Harberts, Max Kusch, John O’Sullivan, Robert Zierold, Robert H. Blick

**Affiliations:** 1 Center for Hybrid Nanostructures, Universität Hamburg, Luruper Chaussee 149, 22761 Hamburg, Germany; jann.harberts@chyn.uni-hamburg.de (J.H.); max.kusch@gmx.de (M.K.); rblick@chyn.uni-hamburg.de (R.H.B.); 2 Department of Physics and Astronomy, University College London, London WC1E 6BT , UK; 3 Material Science and Engineering, College of Engineering, University of Wisconsin-Madison, Madison, WI 53706, USA

**Keywords:** patch clamping, Jurkat cells, human induced pluripotent stem cell-derived neurons, temperature control, electrophysiology

## Abstract

Though patch clamping at room temperature is a widely disseminated standard procedure in the electrophysiological community, it does not represent the biological system in mammals at around 37 °C. In order to better mimic the natural environment in electrophysiological studies, we present a custom-built, temperature-controlled patch clamp platform for upright microscopes, which can easily be adapted to any upright patch clamp setup independently, whether commercially available or home built. Our setup can both cool and heat the platform having only small temperature variations of less than 0.5 °C. We demonstrate our setup with patch clamp measurements at 36 °C on Jurkat T lymphocytes and human induced pluripotent stem cell-derived neurons. Passive membrane parameters and characteristic electrophysiological properties, such as the gating properties of voltage-gated ion channels and the firing of action potentials, are compared to measurements at room temperature. We observe that many processes that are not explicitly considered as temperature dependent show changes with temperature. Thus, we believe in the need of a temperature control in patch clamp measurements if improved physiological conditions are required. Furthermore, we advise researchers to only compare electrophysiological results directly that have been measured at similar temperatures since small variations in cellular properties might be caused by temperature alterations.

## 1. Introduction

In 1780, Luigi Galvani set the cornerstone for modern electrophysiology by accidentally discovering electrical processes within living creatures as electrical currents evoked the movement of frog legs [1]. Two centuries later—in 1976—Neher and Sakmann made a Nobel’s prize-winning breakthrough when they improved the sensitivity of their measurements to such an extent that they were able to measure currents from individual ion channels [2]. Since then, Neher and Sakmann’s patch clamping technique added numerous valuable aspects to our understanding of biological matter and contributed in thousands of publications [3,4]. Today, we know that our entire nervous system is based on the transmission of electrical as well as chemical signals on a single-cell scale, and patch clamping helped fundamentally in understanding the role of many different ion channels in the context of physiological and pathophysiological functions [5,6,7]. In particular, Na^+^, K^+^, and Ca^2+^ voltage-gated ion channels play an important role in the nervous system, and the accurate apprehension of the electrical signaling could even render a direct connection between the nervous system and a computer [8,9]. To address high throughput applications such as drug screening, automated patch clamping and planar patch clamping represent useful extensions to the conventional technique [10,11,12]. However, manual patch clamping is not replaceable at all, since flexibility and data quality are unmatched [13]. In addition, in vivo (automated) patch clamping offers the opportunity to study electrophysiology in living animals—a setting as close as it can get to nature—but such applications are even more challenging due to more complex setups and low giga-seal probabilities [14,15]. For obvious ethical reasons, in vivo patch clamping in humans is out of the question. Thus, patch clamp studies involving the human organism are limited to in vitro experiments with human cell lines or cells derived from human stem cells. However, many experiments to determine electrophysiological properties of suchlike human cells are performed at room temperature (RT) [16,17,18,19,20,21,22]. Thus, all electrophysiological processes within these studies operate significantly below the natural body temperature of about 37 °C. It is worth noting that many biological processes inside the animal’s/human’s body are temperature dependent, and organs like the brain are extremely sensitive to temperature fluctuations; for instance, a core body temperature of about 20 °C would quickly lead to death [23,24]. A patch clamp setup allowing for electrophysiological measurements at controlled temperatures would be of great interest to researchers in order to shed light onto temperature depending cell processes and functions [25]. Unfortunately, commercially available setups from well-established companies, such as “ALA Scientific Instruments” or “Multichannel Systems”, are quite expensive and specific for their dedicated patch clamp platforms. Herein, we present a low-cost, low-tech approach—feasible for everybody and adaptable to every setup independently, whether commercially bought or in-house built—for controlling the temperature of biological samples during patch clamp measurements. In this work, we initially describe the construction details of the microscope inset as well as the corresponding temperature controller. Furthermore, we demonstrate the practical implementation with electrophysiological measurements on Jurkat T lymphocytes (T cells) and human induced pluripotent stem cell-derived neurons (neurons). Aside from basic electrophysiological properties, we compare specific characteristics such as the voltage gating of voltage-gated ion channels and firing of action potentials at RT and 36 °C. Here, we found small influences on non-specifically temperature depending processes confirming the potential need of a temperature control in patch clamp measurements if best biological conditions are required. Furthermore, researchers should only compare their data to the results measured at same temperatures if differences are highlighted compared to literature.

## 2. Materials and Methods

### 2.1. Cell Culture

The T cells were cultured in RPMI 1640+GlutaMAX^TM^-I medium (Gibco) with 25 mM 4-(2-hydroxyethyl)-1-piperazineethanesulfonic acid (HEPES), 7.5% new-born calf serum and 1.2% (w/v) penicillin/streptomycin in T75 cell culture flasks at 37 °C and 5% CO_2_ in air. The cell suspension was diluted with fresh medium every 2-3 days to keep the cell concentration between 0.3 × 10^6^ and 1.2 × 10^6^ cells/mL [26].

The neurons were cultured and differentiated based on the protocol in Reinhardt et al. [27]. Human small molecule neural progenitor cells (smNPCs) were provided in frozen vials from the Max Planck Institute for molecular biomedicine in Muenster, Germany. Further cell culture was conducted on-site. smNPCs were maintained in basic medium (50:50 DMEM/F12:Neurobasal, 1:100 Penicillin/Streptomycin/Glutamine, 1:100 B27 supplement without vitamin A, 1:200 N2 supplement, Life Technologies, Carlsbad, CA, USA) and was supplemented with ascorbic acid (AA, 100 µM, Sigma Aldrich, St. Louis, MO, USA), smoothened agonist (SAG, 0.5 µM, Biomol, Hamburg, Germany), and CHIR (CHIR 99021, 3 µM, Axon MedChem, Groningen, Netherlands). smNPCs were split close to confluency (every 4–5 days) in a ratio of 1:10–1:20. Differentiation into midbrain-dopaminergic neurons (mDANs) was initiated by changing the smNPC growth medium to patterning medium (basic medium supplemented with 100 µM AA, 0.5 µM SAG, 1 ng/mL glial-derived neurotrophic factor (GDNF), and 1 ng/mL brain-derived neurotrophic factor (BDNF), PeproTech, Rocky Hill, NJ, USA). After 6 days, the growth medium was adjusted to maturation medium (basic medium supplemented with 100 µM AA, 2 ng/mL GDNF, 2 ng/mL BDNF, 1 ng/mL transforming growth factor-β3 (TGF-ß3, PeproTech), and 100 µM dibutyryl cyclic-AMP (dbcAMP, Sigma Aldrich)) and the cells differentiated for at least 8 more days (14 in total). The patch clamp measurements were performed after 2–3 weeks after initiation of the differentiation procedure. The cells were kept in a humidified incubator at 37 °C and 5% CO_2_. The growth media were replaced by fresh ones every 2–3 days. All cells were cultured on Matrigel coated 6-well plates and transferred to 35 mm Petri dishes one to two days before the measurements. For passaging/splitting/transferring cells, cells were detached using Accutase^®^ for 10–15 min at 37 °C, centrifuged and subsequently resuspended in the corresponding cell culture medium.

Ethics approval: Jurkat T lymphocytes: cell line, no approval required. Human induced pluripotent stem cell-derived neurons: All experiments were conducted in accordance with the ethical statement in Reinhardt et al. [27].

### 2.2. Patch Clamp Setup

The temperature-controlled inset was installed in a patch clamp setup consisting of a Nikon Eclipse FN1 upright microscope equipped with a non-immersion objective with an extra-long working distance of 11 mm (Nikon CFI TU Plan EPI ELWD 50× N.A. 0.60/W.D. 11.00 mm). Signals were recorded with a HEKA EPC 10 USB patch clamp amplifier using a HEKA red star headstage. The data was processed with a Bessel low pass filter at 2.9 kHz. All measurements were performed with identically fabricated patch pipettes. The borosilicate glass capillary blanks (GB150T-8P, Science Products) were pulled with a Sutter Instrument P-2000 pipette puller (program: three rows with heat = 430, 420, 450; filament = 5, 5, 5; velocity = 70, 70, 70; delay = 200, 200, 200; pull = 0, 0, 0) and subsequently heat polished (CPM-2, ALA Scientific Instruments). The diameter of the pipette tip was about 950 nm, which resulted in resistances of 3–5 MΩ. Pipettes with similar properties prepared with a different pipette puller would be suitable as well. Pipette capacitance and series resistance were automatically compensated.

### 2.3. Electrophysiology

The patch clamp solutions for the T cells were adapted from Partida-Sanchez et al. [28]. The pipette solution consisted of 140 mM KCl, 2 mM MgCl_2_, 1 mM CaCl_2_ and 2.5 mM ethylene glycol tetraacetic acid (EGTA) and is buffered with 10 mM 4-(2-hydroxyethyl)-1-piperazi-neethanesulfonic acid (HEPES). The pH was adjusted to 7.3 with KOH. The bath solution consisted of 140 mM NaCl, 5 mM KCl, 2 mM MgCl_2_, 2 mM CaCl_2_, 5 mM glucose and was buffered with 10 mM HEPES, adjusted to pH 7.4 with NaOH.

For neurons, the patch clamp measurements were performed using a bath solution consisting of 140 mM NaCl, 2.4 mM KCl, 1.3 mM MgCl_2_, 2.5 mM CaCl_2_, 10 mM HEPES and 10 mM D-glucose. The pH-value was adjusted to 7.4 using NaOH. The corresponding pipette solution consisted of 125 mM potassium-gluconate, 10 mM NaCl, 1 mM EGTA, 4 mM MgATP, 10 mM HEPES and 10 mM D-glucose. The pH-value was adjusted to 7.4 using NaOH [27].

Resting membrane potential was measured at zero holding current. Membrane capacitance was determined from the patch clamp software. Membrane time constant was identified by an exponential fit of the membrane potential after a small hyperpolarization. The voltage gating of the T cells was measured by applying a voltage-ramp with a slope of 80 mV/100 ms and determined by the voltage-level showing an increasing membrane current. Action potentials were evoked by stepwise current injections. The data collected at RT and 36 °C originates from at least two independent patch clamp sessions for each cell type. The data was analyzed using C++ programming language. The plots were prepared with Origin (v. 2018). The error bars are the standard error of mean if not stated otherwise. Statistical analysis via ANOVA with post hoc Turkey’s test is given in the supporting materials (*cf.* Table S1).

## 3. Construction of the Temperature-Controlled Patch Clamp Inset

**Figure 1:**
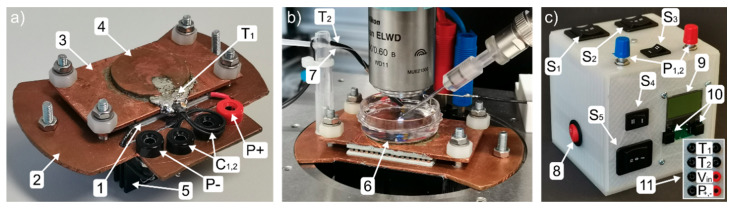
(**a**) Temperature-controlled sample holder: the sample holder consists mainly of a Peltier element (1), fixed between a lower copper plate (2) and an upper copper plate (3), including a copper disc (4), to fit a 35 mm Petri dish. A heat sink (5) is mounted to the lower plate and a thermometer, T_1_, soldered to the copper disc. The Peltier element is electrically connected via P+ and P−. The thermometer is contacted with the two connectors, C_1,2_. (**b**) Sample holder inside the patch clamp setup: The bottom plate fits into the microscope table, which has enough space underneath to accommodate the heatsink. Thermometer T_2_ is used to monitor the temperature of the bath solution inside a 35 mm Petri dish (6). Attached to thermometer T_2_ is the feed line (7) of the fluid compensation system. (**c**) 3D printed housing for the controller parts: power switch (8), switches S_1_, S_2_, and S_3_, potentiometers P_1_ and P_2_, display (9), buttons to change display settings (10), connectors on the backside (11).

The detached microscope inset is shown in Figure 1a. The central component is a Peltier element (1). It is sandwiched between a lower (2) and an upper (3) copper plate to create a temperature gradient between these copper plates. Conventional thermal paste is used to increase the heat transfer and four screws firmly press the components together. The copper disc (4) on top of the upper plate is designed in such a way, that it fits into the bottom of a standard 35 mm Petri dish to ensure maximum heat transfer between cell sample and the upper copper plate. A thermometer T_1_ is integrated into the copper disc in order to monitor the temperature next to the Petri dish. The copper disc and the thermometer are held in place by solder, which ensures good thermal connection between the thermometer and the upper plate. The Peltier element is contacted via banana plugs P+ and P−, and the thermometer is connected by two inner connectors (C_1,2_). Though our sample holder is designed primarily to adjust the temperature above RT, it also allows for the cooling of the bath solution temperature below RT. This feature is, for instance, useful during patch clamp measurements to slow down temperature-dependent processes that are too fast for the standard detection methods and thus enables to resolve recordings beyond the amplifiers time resolution [29]. When the inset is used for cooling below RT, an additional heat sink (5) is required, because the Peltier element has to transport heat from the upper to the lower plate. The performance of this heat transfer process depends mainly on the ability of the lower plate to dissipate the transported heat to the environment. In order to support heating and cooling, two separate controllers for the Peltier element have been used. However, when only the heating of the samples is intended, no heatsink is necessary.

In this study, we designed the lower plate to fit into the microscope platform of a Nikon FN1 microscope shown in Figure 1b. From the backside, the thermometer, T_1_, and the Peltier element are connected. A second thermometer, T_2_, is dipping into the bath solution inside the 35 mm Petri dish (6) to monitor its temperature directly. The different configurations regarding the use of T_1_ and T_2_ are described in the results section later on. In order to compensate for the evaporation of the bath solution in long-term measurements, an automated syringe pump (Aladdin AL-1000) connected through a rubber hose to a thin plastic tube (7) hanging into the bath solution has been used. The necessary refill rates using distilled water have been determined to be 250 and 750 µL/h at RT and 36 °C, respectively, and perfusion was applied using a small continuous flow.

**Figure 2:**
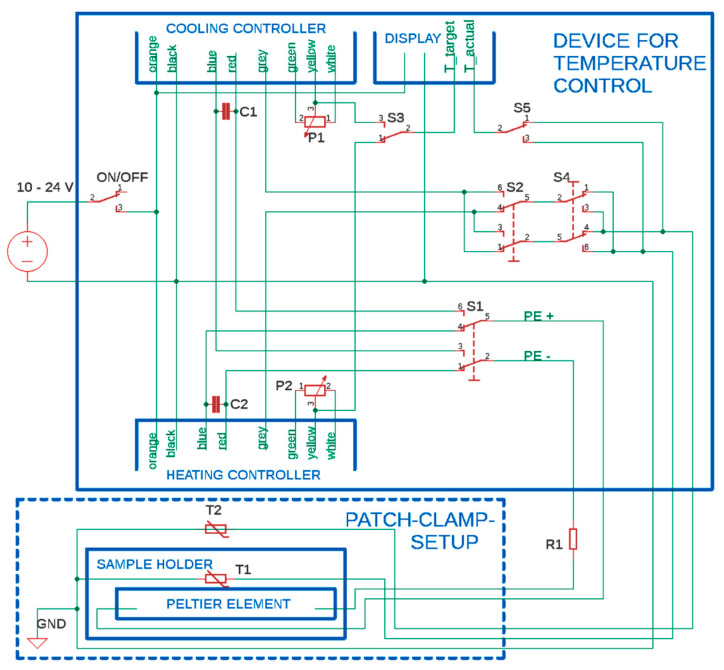
Top: Circuit inside 3D printed housing. Bottom: Microscope inset. P_1_, P_2_: Potentiometer for temperature adjustment. T_1_, T_2_: Thermometers. S_1_, S_2_, S_3_: Switching between heating and cooling. S_4_: Thermometer selection for the controllers. S_5_: Thermometer selection for the display. R_1_, C_1,2_: Low-pass filter and current matching. (Appendix A).

Both controllers for heating and cooling, a display to read out the thermometers, two potentiometers to regulate the temperature, and several switches to choose the operation modes have been built into a 3D printed housing shown in Figure 1c. The functions of the switches are explained in combination with the circuit description in below. The connectors for the power supply, the Peltier element, and the thermometers are placed on the backside. Detailed specifications of the utilized parts can be found at the end of this study, and the 3D model of the housing is made freely accessible online (IEEE dataport, http://dx.doi.org/10.21227/zj2v-9c32). The electrical circuit to connect all parts is shown at full length in Figure 2. The upper blue box displays the electronics inside the 3D printed housing. Both microcontrollers, to drive the Peltier element for heating and cooling, feature their own potentiometer (P_1,2_) to set the target temperature. The electrical circuit design allows for the use of thermometer T_1_ for feedback to the controller and the second thermometer, T_2_, to show the bath temperature on the display. Since both thermometers have to be connected to a microcontroller in order to work properly, both controllers are permanently turned on. Switches S_1_, S_2_ as well as S_3_ are used to change between heating and cooling mode. The switch S_4_ can be used to connect thermometer T_2_ instead of T_1_ to the currently used microcontroller. Last, switch S_5_ is used to select thermometer T_1_ or T_2_ to be shown on the display. The lower dash-lined box contains the parts of the patch clamp inset with the Peltier element and the thermometers T_1_ and T_2_. Note, the capacitors C_1,2_ and the resistor R_1_ form a low-pass filter. It reduces the electromagnetic noise of the microcontrollers that would otherwise disturb the sensitive patch clamp measurements. A small remaining noise in the measurements is removed in data post-processing with a digital filter. Furthermore, the resistor R_1_ also limits the current through the Peltier element to keep the voltage constantly above the minimum of 10 V needed to operate the microcontrollers. Despite the additional resistor, the Peltier element still used more than 1 A of current, which comes along with a notable power drop across the resistor R_1_ leading to a significant heating of the adjacent components. Therefore, the resistor has to be cooled sufficiently and we decided to set up it outside the 3D printed housing. However, the integration of this part into a 3D printed housing can be realized by adding additional ventilation, such as a CPU cooling fan into the housing design.

## 4. Results and Discussion

**Figure 3:**
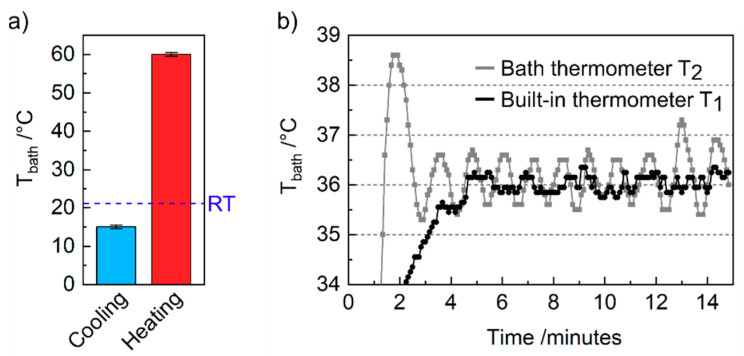
Regulation of the temperature in a 35 mm Petri dish. (**a**) Maximum cooling and heating capability using a 3 A power supply and a passive heat sink. Error bar is ±0.5 °C. (**b**) Temperature course of the bath solution during heating to 36 °C using the bath thermometer and the built-in thermometer as feedback line for the controller, respectively. The temperature was measured with the bath thermometer T_2_.

At first, the maximum cooling and heating performances of the custom-made inset using bath solution in a 35 mm Petri dish have been investigated. We achieved a minimum temperature of 15 °C and a maximum temperature of 60 °C, as illustrated in Figure 3a. These upper and lower temperatures are generally limited by the maximum current output of 3 A from the power source (Laboratory PS-303D) and the capability of the passive heat sink to dissipate the produced heat and thus cool the Peltier element, respectively. Specifically, the upper limit is restricted primarily by the maximal current we used. The lower limit is especially limited at high currents due to ohmic (Joule) heating of the Peltier element itself. Here, in addition to the current induced Peltier effect, the current applied to the Peltier element also produces a considerable amount of heat. Therefore, the cooling of samples relies solely on the Peltier effect and proper heat dissipation from the bottom plate. Since only a passive heat sink has been used in our setup, symmetric cooling and heating from room temperature is not possible. However, symmetric behavior could easily be achieved by improving the cooling performance by using an active water-cooled heat sink, which then replaces the air-cooled finned heat sink.

Then, we investigated the response of the fluid temperature over time when using the built-in thermometer T_1_ and the bath thermometer T_2_ as feedback line for the Peltier heating. Using T_1_ for feedback has been proved to be the better option for temperature control in terms of temperature stability illustrated in Figure 3b. The graph displays the time dependency of the bath solution’s temperature in both configurations during heating from RT to a set point temperature of 36 °C. By utilizing the bath thermometer, an initial temperature overshoot is observable within the first three minutes (gray line), which could be critical for sensitive biological samples. In contrast, a built-in thermometer feedback leads to a smooth approach to the desired temperature (black line). Furthermore, using T_1_ instead of T_2_ reduces the temperature variation from about ±0.5 to ±0.25 °C. The worse response using T_2_ as a loop back signal can be explained by poor heat conductivity of the plastic Petri dish, which inhibits a sufficiently quick response to temperature changes of the sample holder. Using dishes with better heat conductivity—e.g., glass or metal—would reduce the response time. It is worth noting that, when T_1_ is used as a feedback thermometer during sensitive electrophysiological measurements, a calibration of the temperature offset between desired bath temperature and actual value measured at T_1_ is inevitable. In our setup, we determined an offset of approx. 10 °C between T_1_ and T_2_. However, if small (periodic) temperature deviations of ±0.5 °C can be tolerated, a sufficient offset-free temperature control can be achieved after heating up by using S_4_ to connect thermometer T_2_ to the currently used microcontroller. It is worth noting that in both measurements, T_2_ has been used to read out the temperature of the fluid.

Finally, we used the previously described setup to compare—as a proof of concept—the characteristic electrophysiological properties of T cells and neurons at RT and 36 °C. The latter one represents a naturally occurring biological system as it is close to the natural human body temperature while still having a 1 K safety zone to pyrexia. It is worth noting that the investigated parameters are not explicitly known as temperature dependent such as thermoreceptors [30], yet we found a noticeable influence of the temperature. The temperature was maintained using thermometer T_1_ as feedback loop for the Peltier element while observing the correct temperature on the display using thermometer T_2_.

**Figure 4:**
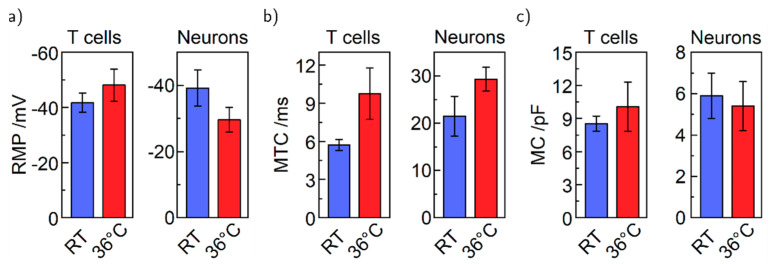
Basic electrophysiological properties of T cells and neurons at RT and 36 °C, respectively. (**a**) Resting membrane potential. (**b**) Membrane time constant. (**c**) Membrane capacitance. T cells: n (RT) = 11, n(36 °C) = 7. Neurons: n (RT) = 8, n (36 °C) = 7.

First, we investigated passive membrane properties that can be determined with both cell types—namely the resting membrane potential (RMP), the membrane time constant (MTC), and the membrane capacitance (MC) shown in Figure 4a–c. The RMP increases for T cells from −41.7 to −48.1 mV and decreases for neurons from −39.2 to −29.6 mV with higher temperature. The MTCs show an increase depending on the temperature for both cell types (from 5.7 to 9.7 ms and from 21.5 to 29.3 ms), while temperature has in principle no effect on the MC of 9 and 6 pF, respectively. Formerly mentioned changes in RMP could be explained for T cells by an increased cellular activity at physiological temperatures, leading to higher RMP values. For the neurons, higher cellular activity might mean that adenosine triphosphate (ATP) is consumed faster at 36 °C compared to RT. A lack of sufficient APT could result in a lower RMP due to ATP-dependent Na^+^ and K^+^ pumps in neurons [31]. For the MTCs, higher temperature raises the probability of open ion channels, which would result in an increased time constant [32]. The capacitance of the membrane is defined predominantly by the surface area of the cell membrane, a measure for the cell size. Therefore, temperature and accordantly the cellular activity have no direct influence on the membrane capacitance. However, all values are still within the error in accordance with the literature [27,33,34,35,36,37]. Only the capacitances of the neurons differ from literature indicated by a smaller value. The reduced MC can be explained by a smaller cell surface area caused by replanting shortly before the experiments (*cf.* methods).

**Figure 5:**
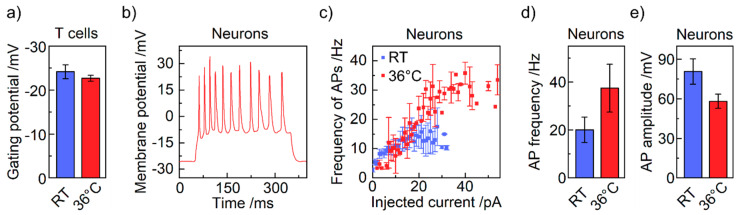
Specific electrophysiological properties. (**a**) Gating threshold of voltage-gated ion channels in T cells at RT and 36 °C. (**b**) Exemplary patch clamp recording of action potentials (APs) at 36 °C during current injection from 50 to 350 ms. (**c**) The firing frequency of APs depending on the injected current for RT and 36 °C of specific cells. The error bars are standard deviation. (**d**) Average maximal firing frequency of action potentials at RT and 36 °C weighted of n cells. (**e**) Average height of APs at maximal firing frequency at RT and 36 °C. T cells: n (RT) = 11, n (36 °C) = 7. Neurons: n (RT) = 8, n (36 °C) = 7.

Second, we tested for cell specific properties in T cells and neurons. For T cells, the gating potential of voltage-gated ion channels is shown in Figure 5a. Voltage ramps from −65 to +15 mV have been applied to the cell. The threshold voltage was defined at the increase in the measured current indicating the opening of the ion channels (*cf.* Supplementary Material Figure S1). The gating potential is generally in accordance with the literature [36,38]. The small shift of the threshold from −23.3 to −25.0 mV—an earlier onset—is reasonable, since most proteins have their peak activity at physiological temperatures rather than at RT and the change in channel-conformation that leads to opening of the channels happens faster at 36 °C than at ambient temperatures [39,40]. For neurons, we tested the capability to fire action potentials (APs) after current-induced membrane depolarization to prove the proper electrophysiological function of the neurons [41]. As a result, the neurons show a characteristic firing of APs for both RT and 36 °C [42]. An exemplary trace recorded at 36 °C is illustrated in Figure 5b. Furthermore, we can confirm a linear dependence of the firing frequency on the amount of injected current shown in Figure 5c [34,43]. Note that the maximum firing frequency of a single cell of approx. 30 Hz at 36 °C is higher compared to approx. 15 Hz at RT. This feature of higher maximal firing frequency was reproducible and goes along with a reduced AP height, summarized in Figure 5d,e. The averaged maximal firing frequency increased from 20 to 36 Hz while the corresponding height decreased from 80.8 to 54.2 mV—in general appropriate values for AP heights [20,35,44,45]. The higher frequency can be explained by faster gating ion channels again allowing for a more rapid sequence of depolarization and repolarization of the cell membrane. Note that the membrane channels at 36 °C can react faster to the ion influx during depolarization than at 20 °C; as a consequence, the repolarization takes place already at lower voltages, leading to smaller action potentials. Literature for patch clamping is not consistent since both temperature dependency and temperature insensitivity for AP frequency have been reported for rat and mouse neurons, respectively [46,47]; however, we can report on a sensitivity. Furthermore, processes in optogenetics showed sensitivity to temperature [48,49]. If one would combine patch clamping with optogenetics, our setup could offer a considerable advantage as well.

## 5. Conclusions

We built an easy to adopt, custom-made, temperature-controlled patch clamp platform for upright patch clamp setups priced at approximately EUR 350 in total. The controller in our presented configuration allows for cooling to 15 °C and heating up to 60 °C limited in our configuration by the passive heat sink and the utilized 3 A power supply, respectively. Temperatures are kept steady at the adjusted values with a tolerance of maximal ±0.25 °C. Furthermore, we show the function of the setup in proof of concept measurements with Jurkat T lymphocytes and human induced pluripotent stem cell-derived neurons. We investigated both basic electrophysiological properties, such as resting membrane potential, membrane time constant, and membrane capacitance as well as cell characteristic responses such as the gating of voltage-gated ion channels and the firing of action potentials. Typically, such properties are not considered as particularly temperature dependent or literature is inconsistent in their findings. However, we found shifts of many parameters when measured at RT and 36 °C, respectively. Therefore, our results advise controlling the temperature if improved physiological conditions are required, which we can offer with our custom-built setup. Furthermore, small disparities among different studies might just be caused due to different temperatures during patch clamping, and we strongly advise considering this if results are compared directly.

## Supplementary Materials

The following are available online at https://www.mdpi.com/2306-5354/7/2/46/s1, Figure S1: Illustration to describe the definition of the gating potential in T cells. Voltage ramp from −65 mV to +15 mV (black). Position/potential of the increase (red dashed) in the membrane current (blue), Table S1: Statistical analysis of patch clamp results via ANOVA with post hoc Turkey’s test.

## Appendix A

Heating controller	Quick Ohm QC-PC-C01H-100
Cooling controller	Quick Ohm QC-PC-C01C
Peltier Element	Quick-Cool QC-127-1.4-8.5MD
Display	Quick Ohm QC-PC-D-100
Potentiometer P_1_, P_2_	0–10 kΩ, supplied with microcontroller
Thermometer T_1_, T_2_	10 kΩ NTC, supplied with microcontroller
Switches S_1_, S_2_, S_4_	Arcolectric H1570 VB AAA 250V/AC
Switches S_3_, S_5_	TRU COMPONENTS TC-R13-66C-02 250 V/AC
Resistor R_1_	ATE Electronics RB50 5.6 Ω 50 W 5%
Capacitors C_1,2_	Yageo SE025M4700B7F-1632
