# Supplementary material for: A Temperature-Controlled Patch Clamp Platform Demonstrated on Jurkat T Lymphocytes and Human Induced Pluripotent Stem Cell-Derived Neurons
Source: Bioengineering (Basel). 2020 May 22;7(2):46. doi: 10.3390/bioengineering7020046 (PMC7355542; doi:10.3390/bioengineering7020046)
Supplement: Supplementary file 1 [file bioengineering-07-00046-s001.pdf]

Supporting Material

A temperature-controlled patch-clamp platform demonstrated on Jurkat T lymphocytes and human induced pluripotent stem cell-derived neurons

Jann Harberts [1,†], Max Kusch [1,†], John O'Sullivan [1,2], Robert Zierold [1,*] and Robert H. Blick [1,3]

[1] Center for Hybrid Nanostructures, Universität Hamburg, 22761 Hamburg, Germany
[2] Department of Physics and Astronomy, University College London, London WC1E 6BT, United Kingdom
[3] Material Science and Engineering, College of Engineering, University of Wisconsin-Madison, Madison, Wisconsin 53706, USA
[†] These authors contributed equally.
* Correspondence: rzierold@chyn.uni-hamburg.de

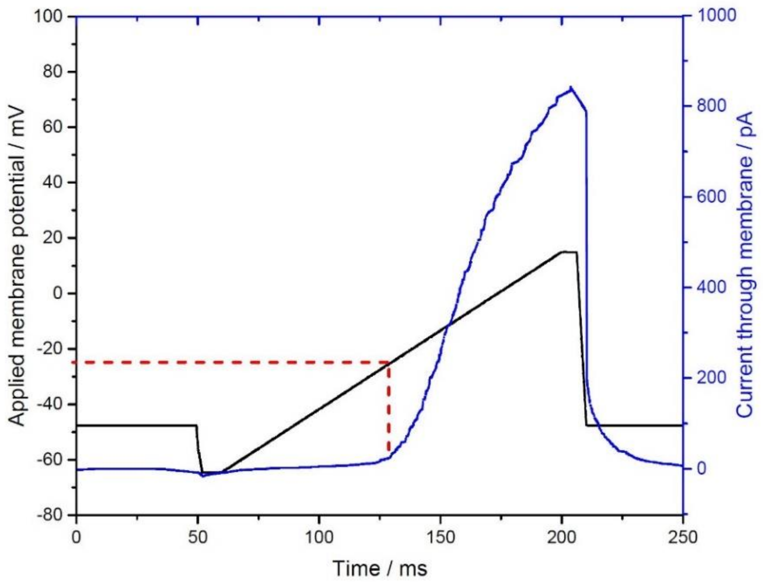

Figure S1. Illustration to describe the definition of the gating potential in T cells. Voltage ramp from -65 mV to +15 mV (black). Position/potential of the increase (red dashed) in the membrane current (blue).

Table S1: Statistical analysis of patch clamp results *via* ANOVA with post hoc Turkey's test.

Cell	Parameter	MeanDiff	SEM	q Value	Prob	Alpha	Sig	LCL	UCL
T cells	RMP	-6.37029	6.41053	1.40534	0.33515	0.35	1	-12.54202	-0.19856
	MC	1.51606	1.95361	1.09747	0.44905	0.45	1	0.00323	3.02889
	MTC	4.02522	1.67845	3.39154	0.02902	0.05	1	0.467	7.58344
	GP	1.49631	2.04625	1.03413	0.4752	0.5	1	0.08412	2.90849
Neurons	RMP	9.59087	6.81989	1.98882	0.18308	0.2	1	0.38282	18.79891
	MTC	7.82678	5.08584	2.17638	0.1478	0.15	1	0.04583	15.60773
	MC	-0.49548	1.61985	0.43258	0.76454	0.8	1	-0.91436	-0.0766
	AP freq.	17.37315	10.8821	2.25778	0.13439	0.15	1	0.72435	34.02195
	AP ampl.	-22.63455	11.44835	2.79604	0.06963	0.1	1	-42.90903	-2.36007
